# Predictors of improvement in disease activity in first hospitalized patients with systemic lupus erythematosus: a multicenter retrospective study of a Chinese cohort

**DOI:** 10.1007/s10067-022-06289-7

**Published:** 2022-07-18

**Authors:** Mei Li, Jun Liang, Wenyou Pan, Lin Liu, Min Wu, Fuwan Ding, Huaixia Hu, Xiang Ding, Hua Wei, Yaohong Zou, Xian Qian, Meimei Wang, Jian Wu, Juan Tao, Jun Tan, Zhanyun Da, Miaojia Zhang, Jing Li, Xuebing Feng, Lihui Wen, Huayong Zhang, Lingyun Sun

**Affiliations:** 1 Department of Rheumatology and Immunology, Affiliated Nanjing Drum Tower Hospital, Medical School of Nanjing University, 321 Zhongshan Road, Nanjing, 210008 China; 2 Department of Rheumatology, Huai’an First People’s Hospital, Huai’an, China; 3 Department of Rheumatology, Xuzhou Central Hospital, Xuzhou, China; 4 Department of Rheumatology, The Third Affiliated Hospital of Soochow University, Changzhou, China; 5 Department of Endocrinology, Yancheng Third People’s Hospital, Yancheng, China; 6 Department of Rheumatology, Lianyungang Second People’s Hospital, Lianyungang, China; 7 Department of Rheumatology, Lianyungang First People’s Hospital, Lianyungang, China; 8 Department of Rheumatology, Northern Jiangsu People’s Hospital, Yangzhou, China; 9 Department of Rheumatology, Wuxi People’s Hospital, Wuxi, China; 10 Department of Rheumatology, Jiangsu Province Hospital of Traditional Chinese Medicine, Nanjing, China; 11 Department of Rheumatology, Southeast University Zhongda Hospital, Nanjing, China; 12 Department of Rheumatology, The First Affiliated Hospital of Soochow University, Suzhou, China; 13 Department of Rheumatology, Wuxi Hospital of Traditional Chinese Medicine, Wuxi, China; 14 Department of Rheumatology, Zhenjiang First People’s Hospital, Zhenjiang, China; 15 Department of Rheumatology, Affiliated Hospital of Nantong University, Nantong, China; 16 Department of Rheumatology, Jiangsu Province Hospital and Nanjing Medical University First Affiliated Hospital, Nanjing, China; 17 Department of Rheumatology, Affiliated Hospital of Jiangsu University, Zhenjiang, China

**Keywords:** Improvement in disease activity, Predictors, Systemic lupus erythematosus, Systemic lupus erythematosus disease activity index

## Abstract

**Objectives:**

To analyze the relative factors of improvement in disease activity (IDA) after first hospitalized treatment based on the systemic lupus erythematosus disease activity index (SLEDAI).

**Methods:**

A total of 1069 adult systemic lupus erythematosus (SLE) patients who were hospitalized for the first time in 26 hospitals in Jiangsu Province from 1999 to 2009 were retrospectively analyzed. SLEDAI decrease ≥ 4 during hospitalization was identified as IDA. Relative factors of IDA were assessed by univariate and multivariate logistic regression.

**Results:**

A total of 783 (73.2%) adult SLE patients showed IDA after the first hospitalization, while the remaining patients (*n* = 286) were in the non-IDA group. The IDA group had higher SLEDAI at admission; fewer patients had SLICC/ACR damage index (SDI) ≥ 1, comorbidities at admission, especially Sjögren’s syndrome, abnormal serum creatinine, and glomerular filtration rate. More patients had mucocutaneous and musculoskeletal involvements, leukopenia, increased C-reactive protein, anti-dsDNA antibody positive, and hypocomplementemia at admission and were treated with methotrexate and leflunomide during hospitalization. After multivariate logistic regression analysis, *SDI* ≥ 1 (*P* = 0.005) and combined with Sjögren’s syndrome (*P* < 0.001) at admission had negative association with IDA. Musculoskeletal involvement (*P* < 0.001), anti-dsDNA antibody positive (*P* = 0.012), hypocomplementemia (*P* = 0.001), and use of leflunomide (*P* = 0.030) were significantly related with IDA.

**Conclusion:**

Organ damage or comorbidities at admission were adverse to SLE improvement. Anti-dsDNA antibody positive, hypocomplementemia, musculoskeletal involvements, and leflunomide treatment had positive association with IDA of SLE.

**Table:** 

**Key Points** • *Organ damage or comorbidities at admission were negatively correlated with SLE improvement.* • *Anti-dsDNA antibody positivity, hypocomplementemia, musculoskeletal involvements, and leflunomide treatment were positively associated with SLE improvement.*

## Introduction

Systemic lupus erythematosus (SLE) is a chronic multi-system autoimmune disease characterized by multiple organ relapsing–remitting disease courses, with a large range of possible clinical and serological manifestations. Despite recent improvements in treatment, the mortality of patients with SLE is still two to twelve times more than that of the general population[1]. Especially, patients with high disease activity are susceptible to organ damage and mortality accrual. Thus, the low disease activity approach to improving disease outcomes drew attention to SLE treatment[2]. To address these issues, “treat-to-target” approaches that focus on achieving low disease activity start to generate; it has been demonstrated to improve patient outcomes across a wide spectrum of medical conditions.

To date, there is no standardized method for defining response to therapy, as disease activity can occur in many organ systems in multiple ways and degrees. Therefore, it is necessary to quantify the decrease and increase in disease activity in a broad spectrum of manifestations. The SLE Disease Activity Index (SLEDAI) is a valid and sensitive gold standard for measuring SLE disease activity, and it has been verified[3]. Based on SLEDAI, the SLE Responder Index-4 (SRI-4) was developed to monitor disease activity, which requires a 4-point improvement in SLEDAI, no new British Isles Lupus Assessment Group (BILAG) “A,” or > 1 new “B” domain score, and no clinically significant worsening (≥ 0.3) in Physician’s Global Assessment (PGA)[4]. However, a limitation of the SRI-4 in clinical practice is that its application of BILAG is time-consuming and complex, and the PGA is subjective judgment varying from person to person. The SLEDAI is reliable and convenient when used by various investigators, and sensitive to the change in a patient’s condition[5].

To evaluate the clinical outcomes of Chinese SLE hospitalized patients, we performed a long-term project to follow up on SLE patients with the earliest clinical and laboratory presentations as well as treatments[6]. In this multicenter study, SLEDAI decreased ≥ 4 during hospitalization was defined as an improvement in disease activity (IDA). The relative clinical characteristics were analyzed according to whether the IDA was achieved, to improve the SLE patients’ therapeutic response and predict clinical outcomes.

## Method

### Study design

Totally, 26 centers participated in this study; all patients fulfilled at least 4 of the revised and/or updated American College of Rheumatology criteria for the classification of SLE [7, 8]. Up to 2015, data of over 2500 cases were documented, which was under the supervision of the Jiangsu Rheumatology Association during the 1999–2009 decade in Jiangsu province, China. A total of 1372 SLE hospitalized patients were enrolled in this study; those with incomplete or loss of related medical records were excluded from the analysis. Patients who were admitted due to many causes other than disease activity were also excluded. At last, 1069 patients were reported in this retrospective study.

### Data collection and definition

The patients’ clinical data on the first admission, including age, gender, diagnostic time, disease duration, disease activity and damage, laboratory tests, organ involvements, and treatments were extracted from the database. SLEDAI score decreased ≥ 4 during hospitalization was considered as IDA. Organ damage was determined by the Systemic Lupus International Collaborating Clinics (SLICC)/American College of Rheumatology (ACR) Damage Index (SDI) [9]. Specific organ involvements were defined: (1) mucocutaneous: mucosal ulceration, skin eruption, cutaneous vasculitis, alopecia, periungual erythema, digital infarcts, angioedema or panniculitis; (2) musculoskeletal: arthritis/arthralgia, myositis/myalgia; (3) neuropsychiatric: headache, acute confusional state, epilepsy, cerebral vasculitis, cerebrovascular disease, demyelination syndrome, myelopathy, aseptic meningitis, cerebellar ataxia, mononeuropathy, polyneuropathy, psychosis, mood disorder (depression/mania); (4) cardiopulmonary: serositis, pulmonary hemorrhage/vasculitis, myocarditis, interstitial lung disease, pulmonary arterial hypertension, cardiac failure, arrhythmia, valvular dysfunction; (5) renal: increased serum creatinine or abnormal glomerular filtration rate (GFR), hypertension (renal related), active urinary sediment, proteinuria, hematuria, biopsy-proven lupus nephritis; (6) gastrointestinal: hepatitis/abnormal liver function, mesenteric vasculitis, peritonitis, lupus gastroenteritis, ascites, malabsorption, protein-losing enteropathy, pancreatitis; (7) hematological: hemolytic anemia, leukopenia, thrombocytopenia. The data were collected anonymously and none of the authors had access to the information after data collection. Normal values for laboratory tests were as follows: white blood cells ≥ 4 × 10^9^/L, hemoglobin ≥ 110 g/L (female) or 120 g/L (male), platelets ≥ 100 × 10^9^/L, alanine aminotransferase (ALT) ≤ 50 IU/L, aspartate aminotransferase (AST) ≤ 50 IU/L, serum albumin ≥ 35 g/L, blood urea nitrogen (BUN) ≥ 7.5 mmol/L, serum creatinine ≥ 133 µmol/L, complement C3 ≥ 0.8 g/L, C4 ≥ 0.2 g/L, anti-nuclear antibody (ANA) ≤ 1: 40, anti-dsDNA antibody negative, anti-Sm antibody negative, rheumatoid factor (RF) < 20 IU/mL, urine protein < 0.5 g/24 h or less than + (urine dipstick testing). Hypocomplementemia was defined as complement C3 < 0.8 g/L and/or C4 < 0.2 g/L. All the antibodies tested were IgG type, ANA was tested by immunofluorescence assay (IFA), and ENA was detected by western blot (WB), and the negativity was defined according to the standard in each hospital.

### Statistical analysis

Statistical analyses were performed using SPSS (Statistical Package for the Social Science) version 22.0 software for Windows. Categorical variables were expressed as numbers and frequencies and analyzed by the chi‑square test. Numeric variables were represented by mean ± standard deviation (SD). For normally distributed data, one-way ANOVA was performed (the Brown-Forsythe test was used if the SDs are not equal). In cases of skewed distribution, the Kruskal–Wallis test was carried out. *P*-values < 0.05 were considered statistically significant.

## Results

The demographics and characteristics of hospitalized SLE patients are listed in Table 1. There were 1069 SLE patients included; 783 patients (73.2%) showed SLEDAI decrease ≥ 4 during hospitalization, which was identified as the IDA group, while another group whose SLEDAI decrease < 4 was defined as non-IDA, which had 286 patients (26.8%). These two groups had no difference in age and gender, while patients with lower weight (*P* = 0.007) tended to attain IDA. The mean SLEDAI scores at the admission of the IDA group and non-IDA group were 16.3 and 11.0, respectively, their difference was significant (*P* < 0.001), and the IDA group’s SLEDAI scores were significantly lower at discharge (4.9 vs 10.4, *P* < 0.001). SDI is an important tool for the evaluation of patients’ almost irreversible organ damages, and we found that the proportion of *SDI* ≥ 1 in the IDA and non-IDA groups were 9.1% and 19.6% respectively (*P* < 0.001).

**Table 1:** Demographics and characteristics of hospitalized SLE patients classified by SLEDAI change (*n* = 1069)

	SLEDAI decrease ≥ 4 *n* = 783 (73.2%)	SLEDAI decrease < 4 *n* = 286 (26.8%)	*P*-values
Age at admission (years)	35.7 (± 11.7)	36.2 (± 11.4)	0.511
Women	726 (92.7%)	266 (93.0%)	1.000
Weight (kg) at admission	55.0 (± 8.6)	56.6 (± 9.1)	0.007
Length of hospital stay	18.0 (± 12.0)	16.7 (± 11.8)	0.124
SLEDAI at admission	16.3 (± 7.2)	11.0 (± 7.5)	< 0.001
SLEDAI at discharge	4.9 (± 5.6)	10.4 (± 8.2)	< 0.001
SDI (≥ 1) at admission	71 (9.1%)	56 (19.6%)	< 0.001
Comorbidities at admission			
Hypertension	98 (12.5%)	49 (17.1%)	0.057
Diabetes	31 (4.0%)	17 (5.9%)	0.182
Sjögren’s syndrome	8 (1.0%)	14 (4.9%)	< 0.001
Infection	7 (0.9%)	7 (2.4%)	0.065
Organ involvements at admission			
Mucocutaneous	551 (70.4%)	173 (60.5%)	0.002
Musculoskeletal	508 (64.9%)	80 (28.0%)	< 0.001
Neuropsychiatric	58 (7.4%)	13 (4.5%)	0.126
Cardiopulmonary	172 (22.0%)	60 (21.0%)	0.802
Gastrointestinal	145 (18.5%)	54 (18.9%)	0.929
Renal	393 (50.2%)	159 (55.6%)	0.128
Hematologic	362 (46.2%)	131 (45.8%)	0.945
Lab tests at admission			
Leukopenia	379 (48.4%)	118 (41.3%)	0.044
Anemia	475 (60.7%)	175 (61.2%)	0.888
Thrombocytopenia	207 (26.4%)	84 (29.4%)	0.352
Elevated transaminases	155 (19.8%)	58 (20.3%)	0.863
Hypoalbuminemia	437 (55.8%)	147 (51.4%)	0.212
Increased blood urea nitrogen	151 (19.3%)	63 (22.0%)	0.342
Increased serum creatinine	47 (6.0%)	33 (11.5%)	0.004
Abnormal glomerular filtration rate	139 (17.8%)	75 (26.2%)	0.003
Increased C-reactive protein	325 (41.5%)	96 (33.6%)	0.020
Proteinuria	409 (52.2%)	154 (53.8%)	0.678
ANA positive	733 (93.6%)	260 (90.9%)	0.139
Anti-dsDNA positive	444 (56.7%)	127 (44.4%)	< 0.001
Anti-Sm positve	282 (36.0%)	90 (31.5%)	0.192
Hypocomplementemia	617 (78.8%)	198 (69.2%)	0.002
Hospitalized treatments			
Steroids	739 (94.4%)	261 (91.3%)	0.069
Anti-malarial drugs	341 (43.6%)	119 (41.6%)	0.578
Cyclophosphamide	344 (43.9%)	133 (46.5%)	0.487
Methotrexate	40 (5.1%)	6 (2.1%)	0.039
Leflunomide	30 (3.8%)	3 (1.0%)	0.017
Mycophenolate mofetil	30 (3.8%)	6 (2.1%)	0.185

*SLE*, systemic lupus erythematosus; *DAI*, Disease Activity Index; *SDI*, Systemic Lupus International Collaborating Clinics/American College of Rheumatology Damage Index

As for the comorbidities at admission, we found that the non-IDA group tended to be combined with Sjögren’s syndrome (*P* < 0.001). The percentage of hypertension, diabetes, and infection had no statistical differences between IDA and non-IDA group.

We collected information on patients’ organ involvements, including mucocutaneous, musculoskeletal, neuropsychiatric, cardiopulmonary, gastrointestinal, renal, and hematologic. We found that the percentage of patients with mucocutaneous (70.4% vs. 60.5%, *P* = 0.002) and musculoskeletal (64.9% vs. 28.0%, *P* < 0.001) involvement was significantly higher in the IDA group, yet no differences were observed in other organ involvements between the two groups.

In addition, we analyzed the difference of the lab tests at admission between two groups, and we found that the IDA group had more patients with leukopenia (48.4% vs. 41.3%, *P* = 0.044), increased C-reactive protein (41.5% vs. 33.6%, *P* = 0.020), positive anti-dsDNA (56.7% vs. 44.4%, *P* < 0.001), and hypocomplementemia (78.8% vs. 69.2%, *P* = 0.002), less with increased serum creatinine (6.0% vs. 11.5%, *P* = 0.004) and abnormal glomerular filtration rate (17.8% vs. 26.2%, *P* = 0.003). While the proportion of the anemia, thrombocytopenia, elevated transaminases, hypoalbuminemia, increased blood urea nitrogen, proteinuria, ANA positive and anti-Sm positive were not significantly different.

With respect to hospitalized treatments, the IDA group had more patients taking methotrexate (5.1% vs. 2.1%, *P* = 0.039) and leflunomide (3.8% vs. 1.0%, *P* = 0.017) instead of steroids, anti-malarial drugs, cyclophosphamide and mycophenolate mofetil.

Through multivariate logistic regression analysis (Table 2), we found that *SDI* ≥ 1 (OR 0.540, *P* = 0.005) and combined with Sjögren’s syndrome (OR 0.183, *P* < 0.001) at admission were adverse to IDA, and musculoskeletal involvement (OR 4.332, *P* < 0.001), anti-dsDNA positivity (OR 1.473, *P* = 0.012), hypocomplementemia (OR 1.785, *P* = 0.001), and leflunomide treatment (OR 4.232, *P* = 0.030) were beneficial to IDA of SLE patients.

**Table 2:** Predictors for the IDA of hospitalized SLE patients

	Univariate analysis		Multivariate analysis	
	Odds ratio	*P*-value	Odds ratio	*P*-value
Weight (kg) at admission	0.980 (0.965–0.994)	0.007	0.988 (0.972–1.005)	0.159
SLEDAI at admission	1.127 (1.100–1.155)	< 0.001	-	-
SLEDAI at discharge	0.890 (0.870–0.910)	< 0.001	-	-
SDI (≥ 1) at admission	0.410 (0.280–0.599)	< 0.001	0.540 (0.350–0.832)	0.005
Comorbidities at admission				
Sjögren’s syndrome	0.201 (0.083–0.483)	< 0.001	0.183 (0.068–0.492)	0.001
Organ involvements at admission				
Mucocutaneous	1.551 (1.170–2.057)	0.002	1.362 (0.995–1.864)	0.054
Musculoskeletal	4.757 (3.535–6.402)	< 0.001	4.332 (3.175–5.911)	< 0.001
Lab tests at admission				
Leukopenia	1.336 (1.016–1.757)	0.038	1.198 (0.880–1.629)	0.251
Increased serum creatinine	0.490 (0.307–0.781)	0.003	0.707 (0.390–1.280)	0.252
Abnormal glomerular filtration rate	0.607 (0.441–0.837)	0.002	0.802 (0.528–1.219)	0.302
C-reactive protein	1.404 (1.058–1.865)	0.019	1.328 (0.969–1.822)	0.078
Anti-dsDNA positive	1.640 (1.248–2.154)	< 0.001	1.473 (1.090–1.990)	0.012
Hypocomplementemia	1.652 (1.219–2.239)	0.001	1.785 (1.269–2.512)	0.001
Hospitalized treatments				
Methotrexate	3.758 (1.138–12.412)	0.030	1.557 (0.616–3.934)	0.349
Leflunomide	2.512 (1.054–5.991)	0.038	4.232 (1.153–15.529)	0.030

*IDA*, improvement in disease activity; *SLE*, systemic lupus erythematosus; *DAI*, Disease Activity Index; *SDI*, Systemic Lupus International Collaborating Clinics/American College of Rheumatology Damage Index

## Discussion

We performed a multicenter retrospective study to explore the relative factors of IDA in first hospitalized SLE patients from a Chinese cohort. We found that SDI (≥ 1) at admission and combined with Sjögren’s syndrome were negatively related with disease improvement, while the musculoskeletal involvement, anti-dsDNA positivity, hypocomplementemia, and leflunomide treatment were positively correlated with the improvement of disease activity. We found the two groups of patients had some different features, so this study was expected to provide a reference for the treatment and therapeutic response of SLE.

We found that patients with higher baseline SLEDAI scores, low complement levels, and positive anti-dsDNA tended to achieve an IDA state under general treatments. Doria et al.[10] and Vollenhoven et al.[11] also showed that patients with higher baseline SLEDAI score, low complement levels, and positive anti-dsDNA had a greater response to belimumab, higher SLEDAI was an independent baseline predictor of response[12]. As we all know, serum anti-dsDNA and complement are not only pathological agents of SLE but also serological markers of SLE disease activity[13, 14]; meanwhile, higher SLEDAI stands for high activity of SLE^13^, so we held the view that patients with higher disease activity likely had a favorable treatment response. It was comprehensible that the higher the baseline level was, the more promptly it dropped. There were clinical trials suggesting that serologically negative patients might not respond as well as serologically active patients to certain agents, which is likely to be associated with autoantibodies’ role in cytokine production and organ damage[14]. Furthermore, Azita et al.[15] found that anti-dsDNA showed a specific association with the attainment of zero scores of SLEDAI. Therefore, the association between serological activity and SLE therapeutic response could not be excluded.

In our cohort, hypertension was the most prevalent comorbidity, and diabetes came the second, yet they had no significant differences between the two groups. SLE with secondary Sjögren’s syndrome (SLE-sSS) was an often-neglected subset in clinical trials, there were few studies about the prognosis of this subset, showing that SLE-sSS had a longer disease duration, more frequent pulmonary involvement, and peripheral neuropathy[16], in agreement with these studies, we found that the non-IDA group had more SLE patients combined with Sjögren’s syndrome. The negative clinical features of SLE-sSS might lead to a worse response to the treatments, while we still need further studies on this matter.

We found that the non-IDA group had more SLE patients with SDI ≥ 1; Zen et al.[17] also held the view that SDI increase was higher in the unremitted patients than the remitted. Gatto et al.[12] found that an SDI score of 0 was associated with low disease activity and remission. Steiman et al.[18] thought there was no difference in the prevalence of major organ involvement in remitted and unremitted patients. Furthermore, we have surprisingly found that mucocutaneous and musculoskeletal involvements were positively associated with patients’ IDA, especially musculoskeletal. The mucocutaneous and musculoskeletal involvements probably responded more sensitively to the general treatments than other organ involvements. We found no difference in neuropsychiatric, cardiopulmonary, gastrointestinal, renal, and hematologic involvements between the two groups, while *SDI* ≥ 1 was negatively associated with IDA, which indicated that in the long-time organ damage, no early organ involvement was associated with bad clinical response; hence, we need to interrupt earlier before the organs involved develop to organs damage so that patients have better clinical responses.

Previous studies indicated that renal involvement predicted SLE poor long-time prognosis[19–21]. While in our cohort, we found that not proteinuria but increased creatinine and abnormal GFR were associated with unfavorable prognosis, which could provide more concrete reference for poor prognosis of SLE treatment.

Leflunomide and methotrexate contributed to IDA when used in combination with general treatment. Leflunomide is a disease-modifying antirheumatic drug commonly used in some autoimmune diseases. It interferes with lymphocytes proliferation by inhibiting the synthesis of pyrimidines and suppresses the production of pro-inflammatory cytokines[22]. Although leflunomide is not recommended for the management of SLE in the EULAR guidelines, several clinical trials conducted in Asian patients have reported its benefits in SLE[22–24]. Zhou et al.[25] found that the leflunomide group had a superior effect on improving joint function compared with the cyclophosphamide group. Apart from it, leflunomide also favored renal function, especially regarding 24-h proteinuria and serum creatinine[26]. Methotrexate is a kind of antifolate that inhibit nucleotide synthesis and DNA replication and is commonly used in rheumatoid arthritis treatment[27]. A meta-analysis indicated that methotrexate was also effective in reducing SLEDAI score and alleviating active arthritis and cutaneous manifestations[28]. Methotrexate appeared to have a steroid-sparing effect and was effective in cutaneous and articular disease[29]. What’s more, 2019 EULAR guidelines for the management of SLE also recommended the addition of methotrexate, especially when combined with skin disease[30]. Therefore, leflunomide and methotrexate could be applied in specific conditions and probably had a surprising therapeutic benefit, especially in the Asian cohort.

The strong point of our study is the large size of the adult SLE cohort from 26 hospitals in Jiangsu Province. It was the first study to explore the relative factors of SLEDAI decrease ≥ 4; some other studies usually use remission (SLEDAI = 0, prednisone ≤ 5 mg/day, without/with immunosuppressants (maintenance dose) and/or antimalarial) or LDAS (SLEDAI ≤ 4, prednisone ≤ 7.5 mg/day, and/or immunosuppressants (maintenance dose)) as the basis of grouping[31]; these group standards were so strict that few patients achieved remission or LDAS and it became rather tough to discover the factors which affect remission or LDAS, while SLEDAI decrease ≥ 4 represented the early response to the treatments and was also very sensitive and convenient to clinical practice; in this way, we could discover more neglected factors which helped us to predict initial SLEDAI decrease. Furthermore, it was reported that early response to the treatments was an important predictor of long-term prognosis[32]. To conclude, SLEDAI decrease ≥ 4 was a potentially helpful and convenient tool in clinical application and research. However, we also have some limitations. Firstly, we were conducting a retrospective research; it was inevitable that there were some incomplete or missing data, which may result in selection bias. Secondly, the included patients were all first hospitalized, so the average score of SLEDAI was much higher than in other studies[11, 33, 34], then the findings of this study were probably only limited to patients with high disease activity, which could not be applied in patients with lower SLEDAI score. Thirdly, this cohort was only from China, and it partly reflected the characteristics of Chinese patients; therefore, the data cannot be extrapolated to other ethnicities. Lastly, this analysis only contained data on patients’ first hospitalization, lacking a long-time follow-up, in which the conclusion could not be used to predict patients’ long-time prognosis and further observational studies were required to assess the applicability of our results. Furthermore, this cohort included patients hospitalized for first time who were treatment naïve or recurrent for any flare; however, we could not distinguish the two kinds of patients for lack of information, whether this would affect our results was not verified and further studies were needed to figure it out. This retrospective study could provide us with relative factors of IDA of SLE patients; this may not necessarily translate to clinical application, but it still needed more clinical trials to be verified; however, this study can give us a hint for clinical practice and deep research.

In conclusion, we searched for the characteristics of patients who had an initial SLEDAI decrease ≥ 4 and found that SDI ≥ 1 and combined with Sjögren’s syndrome were negative for SLEDAI decrease, and musculoskeletal involvement, anti-dsDNA antibody positivity, hypocomplementemia, and leflunomide had positive association with decrease of SLEDAI ≥ 4. This study has provided us with some predictive factors of therapeutic response; therefore, these findings probably assisted clinicians in predicting and improving therapeutic outcomes.

## Data Availability

The data underlying this article are available in the article.
